# *SAXS Merge*: an automated statistical method to merge SAXS profiles using Gaussian processes

**DOI:** 10.1107/S1600577513030117

**Published:** 2013-12-14

**Authors:** Yannick G. Spill, Seung Joong Kim, Dina Schneidman-Duhovny, Daniel Russel, Ben Webb, Andrej Sali, Michael Nilges

**Affiliations:** a Unité de Bioinformatique Structurale, Institut Pasteur, 25 rue du Docteur Roux, 75015 Paris, France; b Université Paris Diderot, Paris 7, Paris Rive Gauche, 5 rue Thomas Mann, 75013 Paris, France; c Department of Bioengineering and Therapeutic Sciences, Department of Pharmaceutical Chemistry, California Institute for Quantitative Biosciences, Byers Hall, 1700 4th Street, Suite 503 B, University of California San Francisco, San Francisco, CA 94158, USA

**Keywords:** SAXS, SANS, data curation, Gaussian process, merging

## Abstract

Small-angle X-ray scattering (SAXS) is an experimental technique that allows structural information on biomolecules in solution to be gathered. High-quality SAXS profiles have typically been obtained by manual merging of scattering profiles from different concentrations and exposure times. This procedure is very subjective and results vary from user to user. Up to now, no robust automatic procedure has been published to perform this step, preventing the application of SAXS to high-throughput projects. Here, *SAXS Merge*, a fully automated statistical method for merging SAXS profiles using Gaussian processes, is presented. This method requires only the buffer-subtracted SAXS profiles in a specific order. At the heart of its formulation is non-linear interpolation using Gaussian processes, which provides a statement of the problem that accounts for correlation in the data.

## Abstract 2

A statistical method to merge SAXS profiles using Gaussian processes is presented.

## 1. Introduction

Small-angle X-ray scattering (SAXS) is a popular experiment that allows low-resolution structural information on bio­molecules in solution to be gathered (Jacques & Trewhella, 2010 ▶; Rambo & Tainer, 2010 ▶; Svergun, 2010 ▶). The SAXS experiment allows for a wide variety of solution conditions and a wide range of molecular sizes. Data collection usually takes between seconds and minutes in a synchrotron facility, or up to a few hours in an in-house X-ray source (Hura *et al.*, 2009 ▶).

The SAXS profile of a biomolecule is the subtraction of the SAXS profile of the biomolecule in solution minus the SAXS profile of the matching buffer. SAXS can be used to study a wide variety of biomolecules, such as proteins, RNA or DNA, and their complexes (Lipfert *et al.*, 2009 ▶; Rambo & Tainer, 2010 ▶), under a variety of experimental conditions. Once this profile is obtained, it can be used for a variety of modeling tasks (Jacques & Trewhella, 2010 ▶; Rambo & Tainer, 2010 ▶; Svergun, 2010 ▶; Schneidman-Duhovny *et al.*, 2012 ▶). It is essential to perform the radial averaging and buffer subtraction steps with high accuracy, as an error at that stage would propagate later on.

The SAXS profile consists of a collection of momentum transfer values (scattering vector) *q*, mean intensities 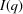 and standard deviations 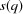. Data collection for a given sample is often repeated a number of times *N* to reduce the noise (or standard deviation) in the SAXS profile by averaging. We consider *N* as the number of points entering the calculation of *I* and *s*, because the variation between repetitions is much greater than that due to radial averaging of a single experiment. Additionally, we suppose that the SAXS profiles were collected at several sample concentrations and X-ray exposure times. Both higher concentration and longer X-ray exposure times can provide more information at higher scattering angles. However, both conditions influence the resulting SAXS profile. At higher concentrations, particle–particle interactions can affect the slope of the very low angle part of the SAXS profile (Glatter & Kratky, 1982 ▶). At longer exposures, radiation damage can perturb any region of the SAXS profile (Kuwamoto *et al.*, 2004 ▶). To remove these unwanted effects it is thus necessary to merge datasets from different experimental conditions. It is the purpose of this method to show that it is possible to do so automatically with minimal user manipulation.

In this article we present the method behind the *SAXS Merge* webserver, a tool presented by Spill *et al.* (2014 ▶) which merges SAXS profiles in a robust, completely automated and statistically principled way. While the method was tested on SAXS datasets, it can also be applied for merging small-angle neutron scattering (SANS) datasets, because the basic equations and methods are similar for the two techniques (Svergun, 2010 ▶). *SAXS Merge* consists of five steps: data clean-up, profile fitting using Gaussian processes, rescaling of each profile to a common reference, classification and detection of incompatible regions, and merging of the remaining data points. The resulting object is a probability distribution function describing the merged SAXS profile. Resulting data consist of the experimental points that are compatible with the distribution, a maximum posterior estimate of the SAXS profile across all experiments along with a credible interval, and estimates of a number of parameters such as the radius of gyration and the Porod exponent.

## 2. Five steps for SAXS merging

*SAXS Merge* consists of five sequential steps: (i) data clean-up, (ii) profile fitting using Gaussian processes, (iii) rescaling of each profile to a common reference, (iv) classification and detection of incompatible regions, and (v) merging of the remaining data points. The first three steps are performed separately on all input SAXS profiles. We now go through each of these five steps sequentially.

### 2.1. Data clean-up

In this step, we remove from input SAXS profiles data values for which the expected value is not significantly different from zero. Let 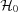 be the null hypothesis of a data point being purely noise-induced. Let 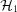 be the alternative that it contains some signal. Then with a type-I error of α, we can perform a one-sample one-sided *t*-test. Let 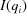 be a mean intensity at momentum transfer 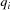, 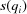 the standard deviation and *N* the number of repetitions of the experiment. Then the *t* statistic is 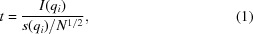
and it has a Student *t* distribution with 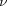 = *N* − 1 degrees of freedom. Since we are performing a large number of tests, we apply the Bonferroni correction by defining α ≡ 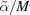 (*M* is the total number of points in the SAXS profile) and choose 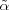 = 0.05 by default. Normality of the noise is assumed, which is reasonable if no parameter varies across the *N* replicates of an experiment.

Points with no or zero standard deviation are discarded. Optionally, points with much larger variances than average are discarded as well. This option is proposed because SAXS profiles have almost constant 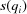 values, except at extreme values for 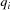 in which case 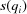 diverges. This behaviour is an experimental artefact, and it is reasonable to remove such points. We therefore calculate the median 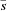 and discard points which have 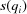 > 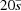.

### 2.2. Profile fitting using Gaussian processes

We have a number of measurements for a SAXS profile, summarized by three sufficient statistics: intensity 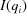, standard deviation 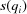 and number of repetitions *N* independent of *i*. The SAXS profile is modelled as the noisy measurement of an unknown smooth function 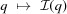 at *M* different data points. A pointwise treatment of SAXS profiles fails because of the high noise and correlation encountered in the measurements. This pointwise treatment would lead to an inconsistent classification [step (iv), data not shown]. It is crucial to account for the correlation between successive points to be able to detect outlier data points in a robust manner. Thus, we first estimate the most probable SAXS profile, which was measured with noise in a given SAXS experiment.

This functional estimation is achieved with the help of the theory of Gaussian processes. Gaussian process interpolation (GPI) is a form of non-parametric fitting which has a straightforward probabilistic interpretation and provides confidence intervals on the functional estimate. Given some data and an automatically adjusting smoothness penalty, GPI provides the most probable function that fits the data. For more information on Gaussian processes, see p. 535 of MacKay (2003 ▶), §13.43 of O’Hagan & Forster (2004 ▶), Rasmussen & Williams (2006 ▶) ▶ and http://gaussianprocess.org.

#### 2.2.1. Likelihood

Define 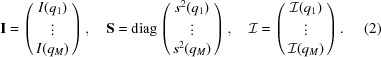


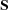
 is the sample covariance matrix, assumed to be diagonal given 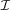. We treat 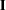 as a measurement with noise of the function 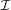 at positions 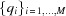 so that 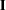 = 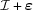 where 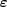 is a vector distributed as a multivariate normal distribution with zero mean and covariance matrix 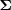. We make the assumption that 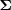 ≡ 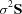, where σ is a proportionality constant that will be estimated in the process. The assumption of a diagonal 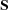 matrix is not entirely correct, as shown by Breidt *et al.* (2012 ▶). However, correlations are expected to be non-zero only between neighbouring annuli (*i.e.* *q* values), and the covariance model we introduce next spans much further than that. This assumption leads to the following likelihood,
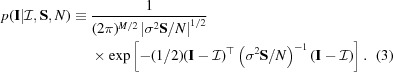



#### 2.2.2. Prior

The likelihood alone does not constrain the vector 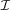, which is still free to vary. However, we believe that the function 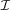 is smooth. This belief is modelled by assuming that the vector 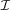 follows a multivariate normal distribution with mean vector 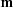 and covariance matrix 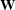 which have been carefully chosen (see below),
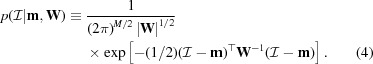
Equivalently, one can say that the function 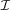 has a Gaussian process prior distribution with prior covariance function *w* and prior mean function *m*.

#### 2.2.3. Choice of *w*

The covariance function determines the smoothness of the Gaussian process. We choose the commonly used squared exponential form, which yields continuous and infinitely differentiable functions. Therefore, this approach is in principle only usable on smooth SAXS profiles,
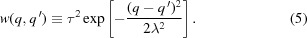
The covariance function has two parameters: 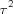 is the variance that the Gaussian process assigns in regions far from any data point; λ is the persistence length of the profile, in units of *q*. With this covariance function, we define 
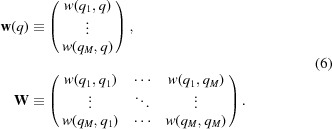



#### 2.2.4. Choice of *m*

Gaussian process interpolation is a non-parametric approach. However, it is possible to incorporate some prior knowledge in the form of a parametric mean function, making the approach semi-parametric. In our case, this way of proceeding has the advantage of providing an estimate of the radius of gyration and other constants. At the same time, the Gaussian process fits the signal in the data that is unexplained by the parametric mean function so that even high deviations from the prior mean function will be followed by the Gaussian process.

At very low angle, the Guinier plot allows for an estimation of the radius of gyration, 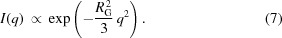
For the higher-angle portion of the profile, Porod’s law is 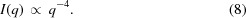
Hammouda (2010 ▶) constructed a smooth function encompassing both behaviours, which we use as a starting point for *m*,
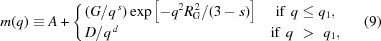


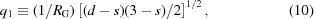


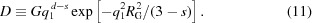
This function has five parameters: *A*, *G*, 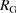, *d* and *s*. Some of them can be fixed to certain values, generating a nested family of parametric functions. For example, setting 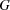 = 0 reduces *m* to a constant function. Setting *d* such that 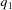 is larger than any input *q*-value reduces *m* to the Guinier model with a constant offset. Finally, setting 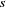 = 0 reduces *m* to the simpler Guinier–Porod model described in the first section of Hammouda (2010 ▶) ▶ (up to a constant offset). Define 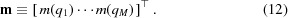



#### 2.2.5. Hyperprior

The parameters arising in the prior mean or covariance functions as well as σ are collectively called *hyperparameters*. In this hierarchical approach we can in turn assign a prior to these hyperparameters. Since our knowledge of their plausible values is rather vague, we give a Jeffreys prior to 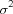 and a uniform prior to the other parameters. However, for the sake of model comparison, parameters are bounded within a finite interval to allow for a normalized prior,
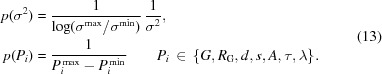



#### 2.2.6. Fitting the SAXS profile

In order to find the best fit of the SAXS profile, it is required to optimize the hyperparameters. Defining 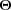 ≡ 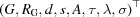 and *D* ≡ 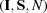, this optimization can be achieved by maximizing 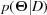 with respect to 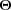. With the help of Bayes’ rule, we obtain
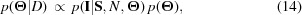
where 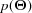 is given in equation (13) and 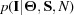 is called the marginal likelihood,
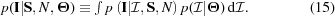
Since both the likelihood [equation (3)] and the prior [equation (4)] appearing in this integral are multivariate Gaussian distributions, it is possible to give an analytical expression of the marginal likelihood,
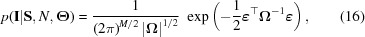
with 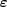 ≡ 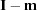 and 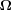 ≡ 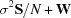.

#### 2.2.7. Obtaining functional deviates

To make predictions of 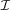 at a new point *q* we average over all possible values for 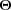, weighted by their posterior probability,
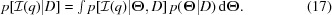
Let us examine the two terms appearing in this last integral. The posterior probability density of the hyperparameters 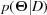 was already encountered in equation (14).

The remaining term, 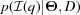, is the posterior predictive probability density of a new noise-free observation given the hyperparameters. It is called posterior predictive because it allows new values of the SAXS profile given the noisy observations to be predicted. Since the function 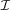 has a Gaussian process prior and a multivariate normal likelihood, the posterior distribution for 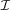 is also a Gaussian process, with mean function 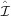 and covariance function 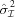 given by
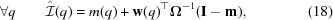


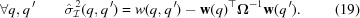
These equations arise from the fact that the vector 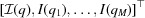 has a multivariate normal distribution with mean vector 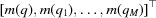 and covariance matrix 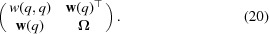
The distribution for 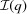 then results from the conditioning of the multivariate normal distribution on the observed values,
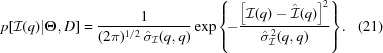
Note that it is also possible to generate random functional deviates from the posterior predictive distribution. If *k* points are wanted for each functional estimate, one can draw them from the multivariate normal distribution with mean vector and covariance matrix built, respectively, from the posterior mean function 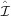 and the posterior covariance function 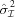 at the values 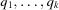.

Although we could in principle perform the interpolation by numerically integrating equation (17) for every value of *q* needed, this approach would be costly in terms of computation power. In fact, two integrals would need to be computed numerically, equation (17) and also the normalization constant of equation (14),
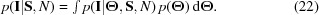
Luckily, as Gibbs & MacKay (1997 ▶) have pointed out, a Laplace approximation of this last integral is a very good approximation because hyperparameters are usually quite peaked around their most probable value. This approach is known as a type-II maximum likelihood (ML-II),
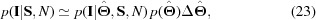


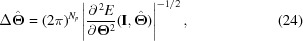


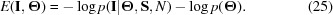


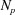
 ≡ 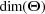 is the number of parameters. 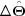 is the phase space volume in which values of 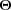 are acceptable given *D*, and is usually small (Rasmussen & Williams, 2006 ▶). This procedure has a considerable practical advantage, since optimization of the hyperparameters then does not need to be performed for each new 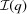 but only once for this dataset. The optimization itself has been described in §2.2.6.

Once the most probable **Θ** has been found, the Laplace approximation gives the normalization constant of 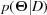, 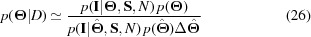
With the additional hypothesis that 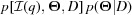 has the same maximum for 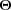 as 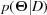 alone, equation (17) becomes
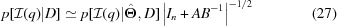


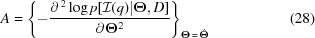


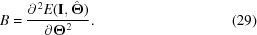
It is also possible to compute the posterior mean and covariance functions averaged over all values of 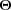,
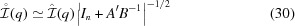


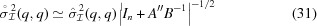


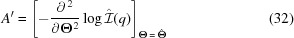


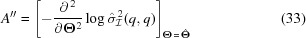



#### 2.2.8. Choice between different mean functions *via* model comparison

Sometimes, the information content of a SAXS profile is so low that the number of parameters in the mean function exceed the number of identifiable parameters of the SAXS profile. In that case, overfitting occurs, and it is preferrable to try a simpler mean function.

The previously presented mean function has five parameters. It has been noted that it generates a nested family of parametric functions when some parameters are held fixed. For globular proteins, *s* can be set to zero, reducing the number of parameters to four. It is also possible to use simpler functions. For example, 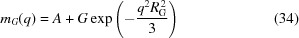
assumes the SAXS profile only contains the Guinier region; it has three parameters. The flat function has one parameter: 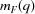 = *A*.

Fitting can be performed using a number of different mean functions. The one that is the most plausible is then selected by model comparison. Suppose 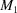 (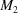) represents the model in which the mean and covariance functions total 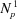 (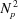) parameters, summarized in the parameter vector 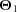 (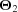). The best mean function is the one which has the highest Bayes factor,
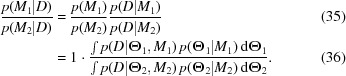
The Bayes factor is the ratio of the evidences if both models have an equal *a priori* probability. As was just discussed, we simplify this assumption further by performing a Laplace approximation of the integral around the maximum posterior set of parameters. This expansion yields
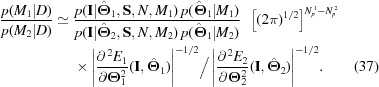
Details of the calculation, along with gradient and hessian of Hammouda’s Generalized Guinier Porod function (Hammouda, 2010 ▶), are given in the supporting information.^1^

### 2.3. Rescaling

Suppose 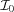 is given, and we want to find the scaling factor γ between 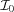 and 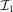, such that the distance between 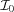 and 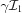 is minimized under some metric. We propose three similar models to rescale the SAXS profiles: normal model, normal model with constant offset, and lognormal model (using the logs of the intensities rather than the intensities themselves). In this section we assume 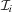 are evaluated at *M* points, and treat 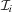 as a vector with *M* entries.

In the normal model we use the squared error loss
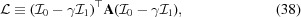
where **A** is a symmetric positive definite matrix. The risk is
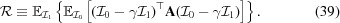
It can be put in the form
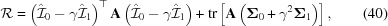
where 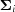 is the covariance matrix of 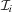. We would like to choose γ and **A** so that the risk is minimal,
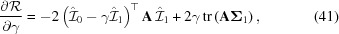


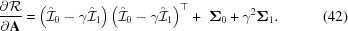
The second equation is a sum of positive matrices, and cannot be zero. Therefore there is no choice of **A** that minimizes the risk. We choose **A** ≡ 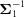. Minimizing the first equation gives the target value for γ,
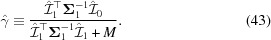
The mean vectors are computed from equation (18) or (30); the covariance matrices from equation (19) or (31).

The normal model with offset has loss function 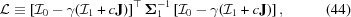
where **J** is a vector of ones. This model leads to the estimates
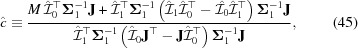


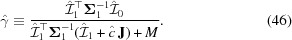
Finally the lognormal model has loss function 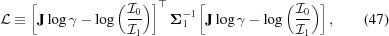
which is defined because the intensities are expected to be positive. The estimate for γ is then
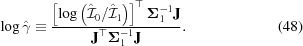
By default, all profiles are rescaled to the last profile, which has usually the widest range.

### 2.4. Classification

To classify the SAXS profiles, it is necessary to rank them. SAXS profiles are ranked as follows. For each profile *i*, we compute 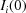 by fitting the Guinier region, and the median 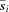 of the errors. We use the median instead of the mean because it is more robust to outliers. The profiles are then ranked by ascending 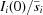. This quantity is expected to increase with either concentration or X-ray dose.

The first profile has the reference status on all intervals that have not been discarded by the first step (*i.e.* as long as its signal-to-noise ratio is sufficiently high). Let 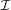 be the candidate profile, and 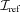 the reference profile, for which we have just derived a distribution in the fitting step. Because correlation has been accounted for in the profile fitting step (§2.2.6), pointwise statistical treatment is sufficient. The SAXS profiles are then compared by using a two-sample two-sided *t* test and regions of disagreement are determined.

We would like to know which measurements of 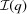 and 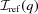 are compatible. We simply assume that each new observation at scattering angle *q* is drawn from a normal distribution with mean 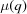 and standard deviation 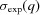, where
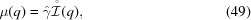


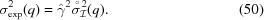


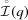
 and 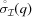 are given by equations (30) and (31) and 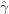 by equations (48), (46) or (43). If no parameter averaging was performed, one can use 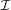 and 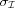 instead of 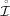 and 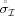 given by equations (18) and (19), respectively.

We then perform Welch’s two-sample two-sided *t*-test at confidence level α (Welch, 1947 ▶). Similar to §2.1, we compute the *t* statistic
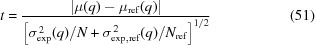
with *N* and *N*_ref_ the number of repetitions of each experiment. The degrees of freedom are given by the Satterthwaite approximation,
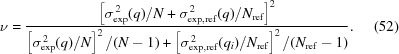
If the *p*-value of this test is smaller than α then the functions are locally different and 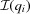 is discarded.

Usually, the second profile spans a wider *q* range, so that comparison with the reference profile cannot be carried out at higher angles. In such a case the remaining portion of the second profile is marked as valid, and becomes the reference. Next, the third profile is compared with the low-angle part of the first profile and with the high-angle part of the second profile. If the third profile spans a wider *q* range than the second profile, its tail becomes the reference for the remaining *q* values, and so on until all SAXS profiles have been compared.

### 2.5. Merging

The merging step simply consists of pooling all compatible data points, keeping track of their origins. Gaussian process interpolation is then performed on this merged dataset. It can then happen that some datasets overlap, leading to multiple intensities for the same *q* values. In that case we discard the points which have the largest standard deviations. This behaviour can be disabled.

## 3. Conclusion

In this article we have developed *SAXS Merge*, a fully automated method for merging SAXS profiles in a robust and statistically principled way. It has been released as both a software package and a webserver, as described by Spill *et al.* (2014 ▶). The required input consists only of the buffer-subtracted profile files in a three-column format (*q*, intensity, standard deviation).

## Footnotes

1 Supporting information for this paper is available from the IUCr electronic archives (Reference: CO5036).
